# Metacognitive therapy for depression

**DOI:** 10.1080/28324765.2024.2308533

**Published:** 2024-01-29

**Authors:** Roger Hagen, Leif Edward Ottesen Kennair

**Affiliations:** a Department of Psychology, University of Oslo, Oslo, Norway; b Department of Psychology, Norwegian University of Science and Technology, Trondheim, Norway; c Research institute, Modum Bad, Vikersund, Norway

**Keywords:** Metacognitions, rumination, metacognitive therapy, depression

## Abstract

Depression is one of the most common psychiatric disorders and frequently accompanied by other mental disorders. There is therefore a need to develop and document new treatments that could have a beneficial effect in treating depression. A new treatment approach to depression that has produced encouraging results is metacognitive therapy (MCT). This paper provides a general introduction to the theory of MCT and especially of how to use MCT in treating depressive disorders. The treatment is illustrated with the clinical case of Andrew, and in the final part of this paper the current evidence for MCT for depression is presented.

## Introduction

Depression is one of the most common psychiatric disorders and frequently accompanied by other mental disorders (Steffen et al., 2020). Cognitive behavioral therapy (CBT) has been viewed as the treatment of choice for depressive disorders, but meta-analyses have shown that the effect has been overestimated (Cuijpers et al., 2013, 2023), and the superiority of CBT over other psychotherapies in treating depressive disorders is not as clear as earlier suggested. It is therefore necessary to develop and document new treatments that could have a greater effect in treating depression than CBT. A new treatment approach to depression that has produced encouraging results is metacognitive therapy (MCT) developed by Adrian Wells (2009). Whilst CBT focuses on the content of thoughts and invites the patient to reality check this content, MCT addresses perseverative thinking processes and metacognitions and does not address the specific content of this processing. CBT is based on Beck’s cognitive model of depression which focuses on the content and validity of negative automatic thoughts, while MCT is based as we shall see below on a specific self-regulatory model of emotional and cognitive processing (Wells, 2009). This paper provides a general introduction to the theory of MCT and especially of how to use MCT in treating depressive disorders. The treatment is described with a fictional clinical case of Andrew (a conglomerate of aspects of patients in our depression trials), and in the final part of this paper the current evidence for MCT for depression is presented.

## The metacognitive model of depression

The theoretical grounding of MCT is the Self-Regulatory Executive Function Model (S-REF model; Wells & Matthews, 1994, 1996). The S-REF model postulates a thinking style called the cognitive attentional syndrome (CAS). In MCT, the CAS is a universal feature of psychiatric disorders and psychological problems and is responsible for prolonging and intensifying distressing emotions. The CAS consists of inflexible self-focused attention, perseverative thinking in the form of worry and rumination, threat monitoring and maladaptive coping behaviors. According to the metacognitive model, different metacognitions control, monitor and appraise the CAS (Wells, 2009). These metacognitions could be divided into positive and negative ones. Positive metacognitions are concerned with the benefits of worry and rumination, while negative metacognitions are concerned with the uncontrollability and danger of thoughts. Negative metacognitions lead to more distress and also to unhelpful behaviors that reduce effective coping (Wells, 2009).

**Figure 1.**
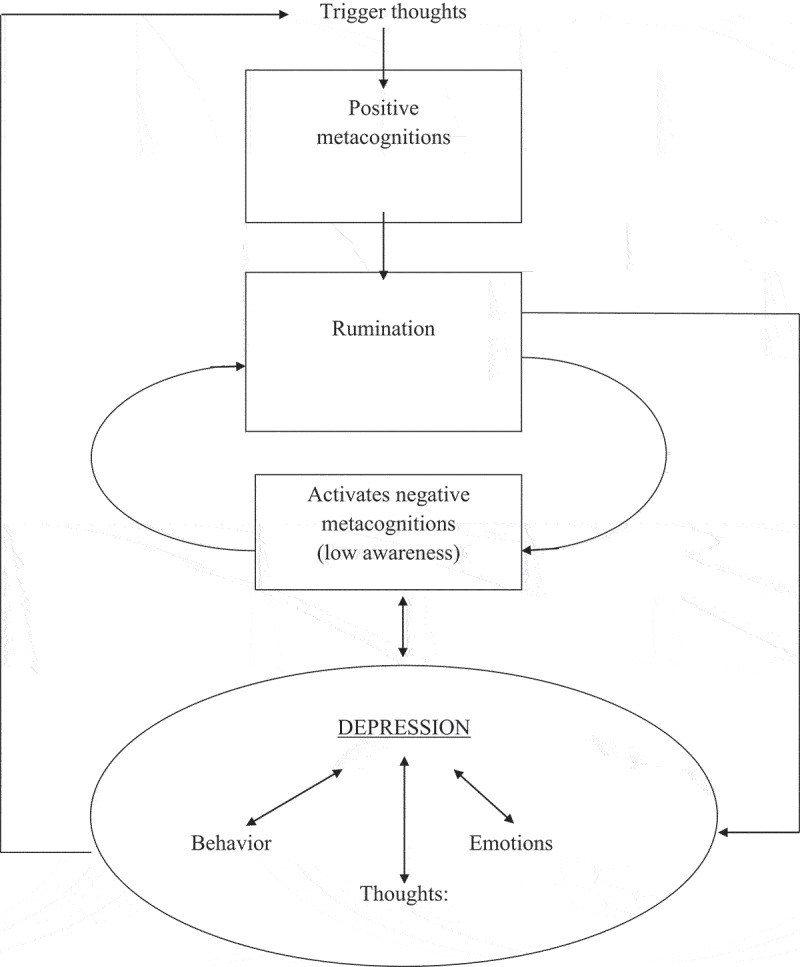
The metacognitive model for depression. (Wells, 2009)

MCT views depression as robustly maintained by rumination, or specifically brooding (Nolen-Hoeksema, 1991, 2000; Nolen-Hoeksema et al., 2008; Treynor. Gonzales & Nolen-Hoeksema). Ruminative thinking is maintained by the patients’ positive and especially negative metacognitions (Papageorgiou & Wells, 2001, 2002). Positive metacognitions related to depression may be exemplified by statements like: ’Analyzing the causes of my sadness will give me an answer to the problem’ and ‘Thinking the worst will make me snap out of it’. Negative metacognitions are activated as the rumination process leads to distress and/or as a result of what the individual learns about depression. Examples of negative metacognitions are: ‘I can’t control my thinking’, ‘My thoughts are caused by my defective brain’, ‘Sleeping more will sort out my mind’ and ‘Thinking like this means I could have a mental breakdown’. The most important factor is the negative beliefs about metacognitions. These metacognitions may further be categorized into two subgroups (Wells, 2009): first, beliefs about the uncontrollability of rumination, including the belief that once one has started ruminating one cannot stop, and second, beliefs about how being depressed and ruminating suggest that there is something (biologically) wrong with one’s brain or that one is somehow psychologically damaged. Patients are not always fully aware that they also have positive metacognitive beliefs, which are the beliefs about the benefits of rumination. To a large degree, such beliefs guide people to choose, albeit more or less implicitly, rumination as a coping strategy. As such, rumination is also partially ego syntonic. The patient believes that rumination will help them cope, solve problems and answer the elusive question of why they have become depressed. Some researchers also believe in these presumed benefits of rumination; however, there is scant evidence for any adaptive value of rumination from either an evolutionary or here and now perspective (Kennair et al., 2017; Nesse, 2019; Solem et al., 2019). The metacognitive model of depression (Wells, 2009) is described in more detail in Figure 1.

The metacognitive model of depression has received support from a range of cross-sectional and longitudinal studies on the effects of metacognitions and rumination on depressive symptoms and disorder. As mentioned above, and a fundamental aspect of the approach, rumination is robustly found to be the major psychological maintaining factor of depression, including exacerbating negative mood, and reducing problem solving (Nolen-Hoeksema, 1991, 2000; Nolen-Hoeksema et al., 2008; Treynor et al., 2003). Further, several studies have considered the metacognitive underpinnings of both rumination and depressive symptoms (Papageorgiou & Wells, 2009; Pedersen et al., 2022; Solem et al., 2016). Typically, it is especially the negative metacognitions that are predictive of rumination and depressive symptoms. This is reflected in the primary focus on negative metacognitions in metacognitive therapy approach described below. While the majority of the data on the MCT model of depression is from Western countries and cultures, there is a growing body of support for the model cross-culturally (Yılmaz et al., 2011).

## MCT for depression

MCT for depression adheres to a particular sequence of different steps across 8–10 sessions (Wells, 2009). The treatment starts with developing a case conceptualization and socializing patients to the model. The therapist and patient work with identifying and labeling rumination episodes with the aim of increasing meta-awareness. The Attention Training Technique (ATT; Wells, 1990) is introduced in the first session to help develop this awareness and as an exercise for gaining more flexible control of their rumination. ATT is thereafter used regularly as a homework exercise during treatment.

MCT progresses by introducing and practicing detached mindfulness, which involves acknowledging the trigger without engaging with it (Wells, 2005) and challenging beliefs about the uncontrollability of rumination. A typical first step is using postponement of rumination as an experiment to evaluate whether rumination is uncontrollable, however managing to use detached mindfulness rather than attempting the maladaptive thought suppression strategy is important here. Other aspects of the CAS are challenged thereafter, including threat monitoring, avoidance and other unhelpful coping behaviors. Positive beliefs about rumination are usually addressed in later sessions, but in some instances, it could be addressed sooner. In the later stages of treatment, focus is turned to relapse prevention, which usually involves challenging residual beliefs and working on a therapy blueprint detailing their new plans for dealing with CAS-related activity (Wells, 2009). The treatment will now be described in more detail, with a fictional clinical case of Andrew.

## The case of Andrew

Andrew is a 28-year male student living alone. For the last 2 years, he has struggled to finish his master thesis. He also reports problems concentrating and is highly self-critical with his writing. This has caused him distress and he is questioning his academic abilities, and he spends a considerable time procrastinating. In addition to this, he is also struggling with finding a romantic partner, since he experiences that many of his friends have started a family and had children. However, he finds it difficult to date as he is struggling with some self-esteem issues and is also doubting his own worth to a future potential partner.

For the last 5 years, Andrew has experienced problems related to depression. He spends a lot of his time ruminating, hoping that he will help him discover why he always feels down ,and what he could do to feel better. He thinks that something is wrong with him since he cannot stop being self-critical. To distract himself from the negative thoughts, he takes long naps and spends most of his time watching TV.

Andrew has gradually lost more and more contact with most of his friends. They used to socialize regularly, but in the last few years he has isolated himself. He feels that his friends have moved on in their lives both in terms of careers and family, while he has accomplished little or nothing. Social activities are, therefore, preferably avoided as they usually trigger more rumination.

### Assessment

Following the diagnostic assessment using the M.I.N.I (Sheehan et al., 1998), Andrew received a diagnosis of recurrent depressive disorder. On the Beck Depression Inventory (BDI; Beck et al., 1961), he had a score of 35, suggesting that he experienced severe symptoms of depression. Andrew did not have any comorbid disorders but reported subclinical symptoms of social anxiety disorder. Andrew also completed a questionnaire related to the metacognitive model called the Major Depressive Disorder Scale (MDD-S; Wells, 2009). The MDD-S assessed how much time is spent ruminating in the last week and how disabling is has been. In addition, the MDD-S assesses the patient’s positive and negative metacognitions, along with the use of avoidance (e.g. interests, social activities, making decisions and getting on with work) and maladaptive coping behaviors (e.g. resting more or using alcohol to cope with depression). The MDD-S is also administered in every session, allowing the therapist to monitor CAS-activity and treatment progress.

### Case formulation

In MCT, the first step in therapy is always to generate a case formulation in collaboration with the patient. With Andrew, the metacognitive model of depression (see Figure 1) was completed using the case formulation interview (Wells, 2009). Andrew was first asked what triggered his rumination and what thoughts followed thereafter: ‘*What was the first thought you had that made you feel depressed? What thoughts followed next? How much time did you spend ruminating?’*. Andrew was then asked questions related to his depressive symptoms: ‘*What feelings and symptoms did you experience when you ruminated, and what did you end up thinking?’* It is also important to assess which strategies patients use to cope with their depression, therefore questions are asked about how it affected his behavior, like: ‘*What do you do to cope with your depressive symptoms?’*

The remaining case formulation incorporates socialization elements as patients are asked questions about their efforts to reduce rumination, as rumination usually is associated with making them feel worse (Wells, 2009). A common question is ’*It seems like you experience problems with not engaging in rumination, how uncontrollable is it?’* The level of uncontrollability is usually rated on a 0–100 scale. Other negative metacognitions are also addressed such as whether they think that their depression is biological and therefore something that they cannot influence. Finally, positive metacognitions are addressed using questions like ‘*Despite all of the negative consequences of rumination, are there any advantages to rumination? Can dwelling on your feelings help you?’* The level of positive metacognitions is also rated on a 0–100 scale.

The case formulation interview with Andrew found that there were several trigger thoughts linked with his rumination. A typical trigger thought was ‘*What’s wrong with me?’* This trigger was followed by periods of rumination that typically lasted for hours and increased his feelings of sadness. His repetitive rumination usually led to him to conclude that he was a total failure. Andrew tried to cope with his depression by taking naps, distracting himself in different ways, he reduced his activity level and withdrew from social situations. These coping behaviors had a paradoxical effect on Andrew as it caused even more rumination, produced new trigger thoughts and amplified his belief that rumination is uncontrollable.

**Figure 2.**
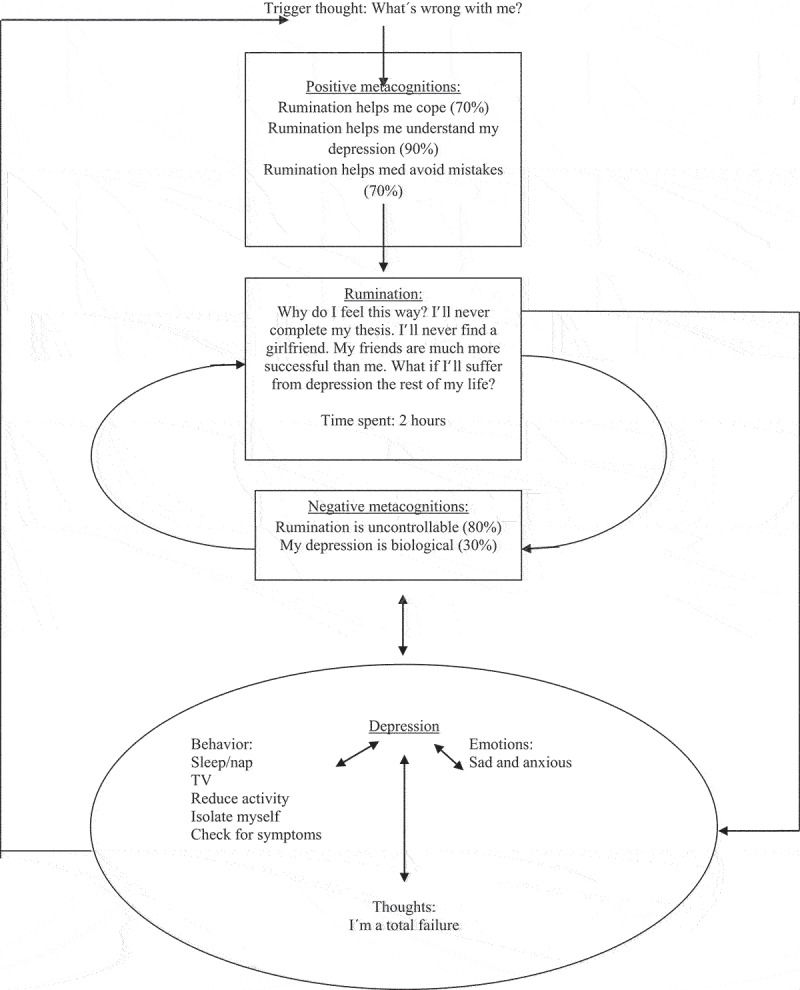
Case formulation for Andrew.

Andrew also reported several positive metacognitions. He believed that rumination could help him find an answer to why he was depressed, and that it could help him cope with his life. According to the metacognitive model, these positive metacognitions will motivate Andrew to ruminate as a reaction to his trigger thoughts, and in combination with his negative metacognitions this will maintain and exacerbate his depressive symptoms (see Figure 2 for a detailed description of the case formulation for Andrew).

### Socialization to the model

After the case formulation, the patient is socialized to a metacognitive understanding of depression (Wells, 2009). The case conceptualization is reviewed together with the patient which prepares the ground for further therapeutic work. Andrew discovered the paradox that he has both positive and negative beliefs about his rumination. He was asked questions about what effect he thought having these contradictory beliefs could entail for him. Similar questions were asked about his coping responses. *Are they helping him? Does his depression improve when he increases his avoidance, inactivity and procrastination?*

Typical questions used in this socialization process include: ‘*How long have you been ruminating about your problems? Has your rumination relieved your depression symptoms? How much longer do you expect it to take? What happens to your sadness when you are distracted from your rumination? How effective have your behaviors been in getting rid of your depression? What happens to your emotions when you increase your activities? What happens to your rumination when you do less? Is rumination a balanced way of viewing your situation? And based on your responses and the model laid out—what do you now think will be important to work on for improving your depression?’* Using such questions, the therapist portrays rumination as an attempt similar to digging yourself out of a hole and helps the patient become more meta-aware rather than focused on reality or depressive thought content.

Andrew agreed that the case formulation fitted well with his experience of depression. He started to understand that rumination maintained his depression, and that the strategies he used to feel better or to cope with his depressive symptoms did not help him feel better but actually made things worse. Even though he felt some shame related to the point that his efforts had been counterproductive, he also felt some hope for improvement and that it was important for him to work on reducing rumination to feel better.

### ATT, detached mindfulness and postponement of rumination

Following the socialization phase, the therapist introduces ATT. As described earlier, ATT is used to help the patient develop awareness and more flexible control of rumination (Wells, 2009). The exercise usually takes around 12 minutes and involves shifting between selective attention, attention switching and divided attention by using an audio clip of 6–9 different sounds. The first 5 minutes are devoted to selective attention as the patient directs attention to individual sounds while resisting distraction by other sounds. The following 5 minutes focus on rapid attention switching while the last 2 minutes with divided attention attempts to process multiple sounds and locations simultaneously.

The rationale for ATT presented to patients is based on that most patients do not recognize that their attention has become habitually locked onto ruminative thinking and have problems interrupting it. (Wells, 2009) Therefore, it should be helpful to become more aware of one’s focus of attention and to strengthen control over it, making it easier to break free of old and unhelpful thinking patterns. It is emphasized that ATT is not used as a distraction from triggers (Wells, 2009). Patients are instructed not to try and stop such trigger thoughts during ATT (see Wells, 2009 for a more detailed description for ATT). Patients often rate their levels of self-focus before and after ATT. The audio clip is shared with the patients, so they can practice the exercise as homework. ATT is thereafter included in every session.

Andrew said that he found ATT difficult the first time he tried it. Trigger thoughts occurred when he experienced difficulties with identifying sounds and also when he lost his focus of attention. He said that he tried to push these away and continue doing the task, but the thoughts were always in the back of his head leaving him uncomfortable. This gave the therapist a segue into the concept of detached mindfulness and an opportunity to reemphasize that awareness of inner events is OK. Further, one reminded the patient that one should continue practicing controlling attention without suppressing trigger thoughts.

A central aspect of MCT is allowing trigger thoughts to come and go without engaging with them. This involves no attempts at controlling or suppressing thoughts or changing one’s behavior in response to them. This is known in MCT as detached mindfulness (DM; Wells, 2005). DM has multiple components requiring the activation of metacognitive knowledge, metacognitive monitoring and control, attentional flexibility and a decentered relationship with thoughts (Wells, 2005). The mindfulness aspect in this regard is related to awareness of thoughts and beliefs and the flexible use of attention. Detachment involves no engagement with the thought (e.g. no appraisal or coping attempts and no initiation of rumination) and dealing with the thought as separate from the self. Different exercises are used to let the patient experience what is meant by DM such as ATT, metacognitive guidance, the free association task, suppression and counter-suppression experiments, and different metaphors (see Wells, 2005, 2009 for more details). Postponing rumination can, as previously mentioned, be used as a technique for testing uncontrollability, but it can also be used as a first attempt toward detached mindfulness (Wells, 2009).

Andrew’s first exercise with DM involved ATT and postponing rumination as homework tasks after the first session. Postponement was framed as a test of both testing whether rumination was uncontrollable but also to see if he was able to not engage in his trigger thoughts. In the second session, he reported that he was successful in postponing rumination at times, but at other times, he found it more difficult and said that he experienced it as habitual behavior. The second session was devoted to DM and challenging uncontrollability beliefs. One exercise used was the free association task. In this task, the therapist said a series of neutral words, for example, green, water, sun, walking, bicycle, holiday, chocolate and so on. Andrew’s task was to let his mind wander freely without trying to control his thoughts and to just observe his mental experiences in a detached manner while listening to the list of words. With this increased ability to observe and not engage with thoughts, the free association task was gradually expanded by dropping in trigger words related to Andrew’s depression. He was specifically asked to treat trigger words in the same way as neutral words. Slowly, Andrew grasped the concept of detached mindfulness and how this was fundamentally different from his normal way of reacting to trigger thoughts with rumination and worry. This task was repeated and fine-tuned until Andrew got a better grasp of DM and how it differs from thought suppression. This was also accomplished by the therapist using a thought-suppression experiment where Andrew was instructed not allow himself to think about something for a couple of minutes. Combining ATT, DM and rumination postponement gave Andrew more control over his rumination and reducing such activity could alleviate his depression symptoms.

### Challenging negative metacognitions

Central to MCT is modifying negative metacognitions, and the most important of which is the belief that rumination is uncontrollable (Wells, 2009). Together with the patient, the therapist looks for examples of exceptions to this: ’*How were you able to postpone your rumination? Can you increase you rumination? What happens to your rumination when you are distracted (e.g. if an emergency occurs)? If rumination is completely uncontrollable, how does it ever stop?’* Therapists can then go on to exercises where the patient tries to increase rumination and then interrupt the process. Other negative metacognitions are often challenged using behavioral experiments and socratic questioning and involve testing whether one can lose control of their rumination, whether ruminative thinking suggests that you are defective, or if depressive thoughts are a sign that you could be losing your mind (Wells, 2009).

Andrew gradually gained more experiences with exercising control of his ruminative thinking. He stated that he became more aware of his typical trigger situations and his responses to them. He still felt that his habitual choice of behavior was to engage in them, but that he made a conscious decision to detach from further thinking and instead focusing on the activity he was engaged in irrespective of what it was. He described that he was ‘tempted’ at times to engage in trigger thoughts but then remembered that the topic is something that he has thought about a thousand times before, and that further processing was likely to be unproductive. He also felt that the therapist had normalized trigger thoughts to a great extent, and that everyday fluctuations in mood were not of great significance. As Andrew was able to reduce his rumination, this was accompanied by less strong beliefs in the uncontrollability of rumination, which gave him hope of recovery.

### Challenging positive metacognitions

When uncontrollability of rumination is dealt with effectively, the focus shifts to positive metacognitions (Wells, 2009). Different strategies can be used for modifying such beliefs. Some questions that can be helpful include: ’*If there are only benefits to rumination, why don’t you ruminate more? If rumination is helpful, why are you still depressed?’* Employing socratic questioning, the patient could become aware of the disadvantages of rumination, in fact using an advantages-disadvantages analysis could be helpful in this respect. The therapist may also challenge the listed advantages. In general, many patients find it helpful when the therapists question the evidence for the patient’s positive beliefs.

Andrew’s positive metacognitions had already started to decrease as a result of his reduced rumination, but he still reported some positive beliefs. One aspect he found difficult was that he sometime wondered if he was ruminating, reflecting or problem solving. Rumination is rarely just repetition of negative thoughts. Most likely, it fluctuates between rumination about past losses and failures, memories of difficult events and worries about the future along with attempts at mentally solving these and even attempts at thinking more realistic or positive. However, all this extended thinking in response to a trigger would be considered as counterproductive according to the metacognitive model. Andrew was therefore encouraged to avoid attempts at categorizing his own thinking and practice DM in these situations. Andrew also tried an experiment where he ruminated one day and compared it with one day without any rumination, to assess the usefulness of rumination. Andrew’s experience was that the day he ruminated he felt much worse and did not get any work done, while he felt better and was much more productive the day he did not ruminate.

### Working with unhelpful coping strategies

Patients with depression often use different coping strategies that backfire and maintain their depression. Metacognitive therapy therefore addresses these behaviors and their associated beliefs (Wells, 2009). A common coping behavior involves overfocusing on one’s symptoms through body scanning and symptom checking. For example, if the patient wakes up with a feeling of low energy, this is attributed as a sign of depression and that one should rest until one feels better. This preoccupation with symptoms causes patients to become more aware of their negative thoughts and bodily symptoms, which maintain their depression. Other typical unhelpful coping behaviors include reduced activity, increased sleep, use of alcohol, avoidance and self-harm. Together with the patient, the therapist explores the effects such monitoring has on depressive symptoms (most likely it reinforces depressive symptoms), and how this activity can be challenged. One way of doing this is by using an advantage-disadvantage analysis and banning such activity.

Andrew had different maladaptive coping behaviors. Together with the therapist, he tried to increase his activity level including social activities and time spent working on his thesis. Related beliefs such as ’*I can’t finish my paper because I’m feeling tired and sad all the time’* were challenged and tested. Andrew experienced that increasing his efforts improved his mood. This was further strengthened when he started to see that his work paid off and as he received positive feedback from his supervisor. Andrew found it difficult to approach his friends at first, and the social interaction triggered the same thoughts as before. However, this faded with repeated experience, and he also found it helpful and normalizing to see that they also experienced everyday struggles with their work and families. Andrew’s attentional focus on symptoms of depression was also discussed in therapy. Together with Andrew, the therapist reviewed the advantages and disadvantages of having such a focus of attention, and whether this symptom checking and body scanning should be discontinued.

### Relapse prevention

As treatment with Andrew approached the latter stages focus turned to relapse prevention. It is important for the patient to see the difference between their old and new ways of dealing with their negative thoughts and depressive symptoms to consolidate and strengthen their new metacognitive plans. A therapy blueprint (summary sheet) is outlined collaboratively and as homework. This blueprint has also been referred to as the ‘old plan/new plan’ (Wells, 2009). The plan specifies the case conceptualization and how the patient dealt with CAS-activity when depressed (left column) along with another column specifying new ways of dealing with depression (the right column). The plan typically includes the patient’s trigger thoughts, examples of ruminative thinking, negative and positive metacognitions, and coping behaviors including attentional focus. The blueprint attempts at explicitly stating what the patient has learned during treatment and can function as a reminder in the follow-up period after treatment. In addition to the blueprint, the final sessions are also used to deal with residual beliefs.

Andrew wrote down several aspects he found important during treatment. In the old plan column, he wrote down that he used rumination to help him cope, as an attempt to understand and solve problems, and to avoid making mistakes. In the new plan column, he wrote ‘*Rumination doesn’t help at all! It makes things worse*’. He also made a note that he could exercise control over rumination, that he should be less self-critical and that trigger thoughts and fluctuations in mood is something that happens to everyone. He also remarked that reducing rumination helped him sleep better. In the left column, he wrote that he used to reduce his activity, while the right column stated that he could still be productive even if he was not feeling great. One of his old strategies was to closely monitor his energy level. Another aspect was that he used to focus his attention inward (his thoughts, energy and mood) but now shifted towards a more external focus of attention. Andrew also wrote that he now made decisions easier than before, without thinking about all the imaginable pros and cons. A closer view of Andrew’s new and old plan is found in Table 1.

**Table 1.** Old plan-new plan for Andrew

Trigger-thoughts I’m afraid to become depressed again. I’m a failure Nobody wants me.	
**Old plan:**	**New plan:**
**Thinking style:** Ruminate about things in order to find an answer. To ruminate about myself will help me cope with my life. Ruminate about my situation and my life in general will help me snap out of the depression.	**Thinking style:** Do not care about what other people think. Postpone rumination, and let the negative thoughts be left alone and not engage them. Do things, instead of just ruminate
**Behaviour:** Isolate myself from others. Sleep more. Try to distract myself from negative thoughts by watching Netflix and TV.	**Behaviour:** Become more social and take more initiative in order to be together with others. Activate myself and have a plan for things to do during the day/week.
**Attention focus:** Occupied of my negative thoughts. Checked my mood and feelings, and whether I could find any sign of depression. Self-critical attention	**Attention focus:** Focus on other things than myself. Focus on things I have achieved in my life. Be in the present moment and reality, instead of being in my thoughts
**Reframe:** Rumination is destructive for me in order to have a better life. In order to cope with my depression I must change my thinking style, my attentional focus and my behavior.	

## Outcome studies for MCT for depressive disorders

Several studies have explored the effects of MCT for depression both individually and in group settings. Below we provide a short review of these studies related to outcome in treating depression. We have divided the studies in open trials and randomized controlled trials related to individual therapy and also summarize shortly the studies done in group settings.

### Open trials

The first study that explored the effect on MCT for depression was a small multiple-baseline study with four patients suffering from recurrent and persistent depression. All included in the trial had previous episodes most with early debut of depression, and the patients had previously received therapy and three out of four were on medication treating depression. The number of sessions varied between 6 and 8. The study reported large and clinically significant improvements for the patients in relation to depressive symptoms, metacognitive beliefs at the end of treatment (Wells, 2009). According to the Jacobson and Truax (1991) criteria at post-treatment three out of four were recovered and one was reliable improvement. The results were maintained both the 3- and 6-month follow-up.

In 2012, a platform trial was published with 12 patients with treatment-resistant depression (Wells et al., 2012). Treatment-resistant depression was defined as not responding to at least a minimum dosage of antidepressive medication taken for at least 6 months and not responding to psychological treatments in the past. The mean number of sessions was 6.5. Dependent on the criterion, the recovery rates varied between 60 and 80%. These were largely maintained at 12-month follow-up. The effect sizes were 1.65 and 2.71 for measures of depressive symptoms from pre- to post-treatment and follow-up (Cohen’s *d*).

An open trial in Norway included 10 patients with major depressive episodes and comorbid disorders (Hjemdal et al., 2017). The patients were given 10 sessions of MCT. According to Jacobson and Truax (1991) criteria, at post-treatment 90% were recovered and 10% improved, while at 6-month follow-up 70% were recovered, 20% improved and 10% had no change. The trial also reported an effect size of 2.89 (Hedge’s *g*) related to depressive symptoms. At the end of treatment, patients were no longer diagnosed with initial comorbid disorders like GAD, panic disorder and social phobia.

Finally, an open trial from Germany by Winter et al. (2019) included 30 depressed patients, 15 with major depressive disorder (MDD) and 15 persistent depressive disorder (PDD). PDD was defined as either failed treatment attempts with anti-depressive medication or CBT. The mean duration of treatment was 16 weeks with one session per week as the norm. The results indicated that 66.7% with MDD and 80% with PDD were recovered at end of treatment and a within-effect size of 3.40 (Cohen’s *d*) for the MDD group. The high change in PDD indicates that causal mechanisms involved may be the same across MDD and PDD, and that MCT addresses them both. The finding was interpreted as promising as it supports the notion that differentiating between different types of depression may not be necessary.

### Controlled randomized trials

The first randomized controlled trial was run in Australia and compared MCT and CBT with 48 patients. They were given 12 sessions in each treatment condition. The effect sizes were moderate to large with Cohen’s *d* of .96 for CBT and 1.12 for MCT at end of treatment. No differences were found between the therapies (Jordan et al., 2014). However, there were more patients with serious comorbid disorders, and therapists had substantially less training in the MCT condition.

Another randomized controlled trial compared MCT with a wait-list condition with 39 patients that suffered from primary depression. The patients received 10 weekly sessions (Hagen et al., 2017). The controlled effect size was Cohen’s *d* of 2.51 for self-reported depressive symptoms with 70–80% recovered at post-treatment and 6-month follow-up. The results were maintained with a 70% recovery rate at 1-year follow-up (Hjemdal et al., 2019) and at 3-year follow-up (Solem et al., 2019). At 3-year follow-up, the patients also showed improved quality of life and had increased return to work or studies.

A larger randomized controlled trial published in 2020 compared the effect of MCT (85 patients) and CBT (89 patients) (Callesen et al., 2020). Up to 24 sessions were given. For self-report, there were significant differences between the groups at post-treatment and 6-month follow-up. In all, 74% were recovered in the MCT condition compared to 52% in the CBT condition, with controlled effect size of 2.06 for MCT and .68 for CBT (Hedge’s *g*). The primary limitation of this study was that only two therapists were involved.

Taken together, these studies of MCT in individual therapy setting find evidence that MCT for MDD is highly effective and indications that the recovery rates are high and show substantially lower relapse rates compared to what is reported for other treatments (e.g. Normann & Morina, 2018). While more research is needed to substantiate this further, MCT for MDD remains highly promising.

### Metacognitive group therapy

The metacognitive theory postulates common causal mechanisms for a variety of mental disorders with a focus more on thinking processes rather than content, which makes it particularly interesting to explore the effect of MCT in a group therapy format.

Papageorgiou and Wells (2015) were the first to publish a study using MCT in a group format for depressed patients. Ten patients with depression who had not responded to both antidepressants and CBT were included and given 12 2-hour weekly sessions and two post-treatment booster sessions. All completed the treatment and 70% were recovered and 20% improved at both end of treatment and 6-month follow-up and an effect size of 2.88 (Hedge’s *g*). The frequency of comorbid Axis-I disorders was also reduced.

This finding was followed up in an open trial by Dammen et al. (2015). Their study included 11 patients with MDD who got 10 weekly 90-minute sessions of group MCT. Groups consisted of five and six participants, which equaled to between 2.5 and 3 therapist sessions per patients. All patients were recovered at post-treatment and 91% at 6-month follow-up. Findings at post-treatment indicated a relief of comorbid axis I and II diagnosis apart from somatoform pain disorder. The patients were followed-up at 1 and 2 years with recovery rates of 70% and 80%, respectively (Dammen et al., 2016).

Recently, Strand et al. (2023) evaluated delivering group MCT for patients with MDD and comorbid disorders in the specialized mental health-care system in Norway. A total of 17 patients were included, and they were given 10 sessions of generic group MCT. The results indicated large effect sizes for Cohen’s *d* for self-reported depression (1.85), anxiety (1.56), interpersonal problems (1.00) and general functioning (.94). Recovery rates from pre- to post-treatment for self-reported depressive symptoms were 35.29% with 47.06% improved and no change of 17.65%. while for anxiety symptoms, the recovery rates were 64.71%, 23.53% improved and no change 11.77%. No patients reported worsening and no patients dropped out of treatment. Each patient received an average of seven therapist sessions.

## Conclusions

The recovery rates for MCT are fairly high, often around 70%, and often remain relatively unchanged over time and for some studies up to 3 years. Studies also show substantially lower relapse rates compared to what is reported for other treatments (e.g. Normann & Morina, 2018). Even though the results are promising, future trials on the effect on MCT for depression should apply RCT design, with evidence-based comparison conditions to assess whether MCT is as effective as other psychotherapies for depression, and more research is therefore needed.
